# High‐Mobility p‐Type Organic Semiconducting Interlayer Enhancing Efficiency and Stability of Perovskite Solar Cells

**DOI:** 10.1002/advs.201700025

**Published:** 2017-04-21

**Authors:** Mingyu Zhang, Jiayu Wang, Liang Li, Guanhaojie Zheng, Kuan Liu, Meng Qin, Huanping Zhou, Xiaowei Zhan

**Affiliations:** ^1^ Department of Materials Science and Engineering College of Engineering Peking University Beijing 100871 P. R. China

**Keywords:** high mobility, interlayers, organic semiconductors, perovskite solar cells, p‐type

## Abstract

A high‐mobility p‐type organic semiconductor based on benzodithiophene and diketopyrrolopyrrole with linear alkylthio substituents (BDTS‐2DPP) is used as a dual function interfacial layer to modify the interface of perovskite/2,2′,7,7′‐tetrakis(*N,N*′‐di‐*p*‐methoxyphenylamine)‐9,9′‐spirobifluorene in planar perovskite solar cells. The BDTS‐2DPP layer can remarkably passivate the surface defects of perovskite through the formation of Lewis adduct between the under‐coordinated Pb atoms in perovskite and S atoms in BDTS‐2DPP, and also shows efficient hole extraction and transfer properties. The devices with BDTS‐2DPP interlayer show a peak power conversion efficiency of 18.2%, which is higher than that of reference devices without the BDTS‐2DPP interlayer (16.9%). Moreover, the hydrophobic BDTS‐2DPP interlayer effectively protects the perovskite against moisture, leading to enhanced device stability.

## 1 Introduction

Recently, solution‐processed organic–inorganic lead halide perovskite solar cells (PSCs) have gained worldwide attention due to their high efficiency and low cost.^1^ Since the first attempt using perovskite as light absorber in photovoltaic cells,^2^ tremendous progress has been made on PSCs with a certified power conversion efficiency (PCE) exceeding 20%.^3^ The impressive efficiency of PSCs is mostly attributed to merits of perovskite, such as broad absorption with large absorption coefficient, ambipolar charge transport with high mobility, as well as long electron/hole diffusion length.[[qv: 1b,2,4]]

In most of the high‐efficiency PSCs, 2,2′,7,7′‐tetrakis(*N,N*′‐di‐*p*‐methoxyphenylamine)‐9,9′‐spirobifluorene (spiro‐OMeTAD) and poly(triarlyamine) (PTAA) have been used as the main hole transport layer (HTL).[[qv: 1c,3a,4f,5]] However, they need be doped with lithium bis(trifluoromethysulfonyl)imide (LiTFSI) and 4‐*tert*‐butylpyridine (tBP) to improve their relatively low hole mobility (10^−4^–10^−3^ cm^2^ V^−1^ s^−1^ in the pristine film). These additives are hydrophilic and accelerate the degradation of devices.[[qv: 4a,5a,6]] To improve device stability and efficiency, various interfacial materials, such as metal nanostructures,^7^ graphene derivatives,^8^ and thin insulating layer,^9^ are used to modify the interface between perovskite and HTL.

Due to the poor thermal stability of perovskite, loss of halide ions will lead to the under‐coordinated Pb atoms or clusters, and these defects may induce charge recombination and decrease the device performance.^10^ Therefore, it is necessary to passivate the trap states of perovskite. Some n‐type organic semiconductors containing nitrogen or sulfur atoms are used to passivate the perovskite defects in inverted PSCs,^11^ since nitrogen or sulfur atoms can provide lone pair electrons to form coordination bonding with under‐coordinated Pb atoms. However, to our knowledge, there have been no reports for p‐type organic semiconductors to serve as the interlayer at perovskite/HTL interface in conventional PSCs.

**Figure 1:**
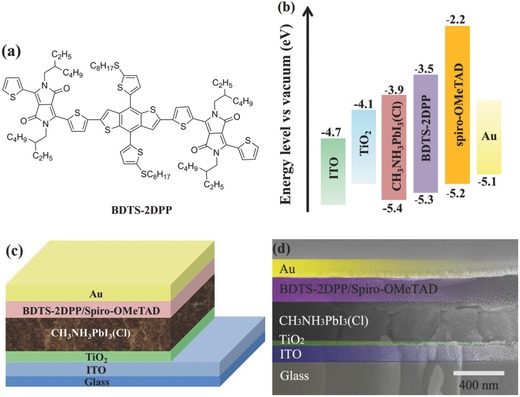
a) Chemical structure of BDTS‐2DPP, b) energy level diagram of PSCs, c) device structure of PSCs, and d) SEM image of cross‐sectional structure of PSCs.

In this work, we demonstrate the first example for p‐type organic semiconductors used as the interlayer at perovskite/HTL interface (BDTS‐2DPP) in conventional PSCs to improve the efficiency and stability. BDTS‐2DPP is based on benzo[1,2‐*b*:4,5‐*b*′]dithiophene flanked with diketopyrrolopyrrole (**Figure** **1**a),^12^ and exhibits good solubility in organic solvents, high hole mobility (>10^−2^ cm^2^ V^−1^ s^−1^), as well as compatible energy alignment with CH_3_NH_3_PbI_3_(Cl), and may efficiently extract holes from perovskite to HTL. On the other hand, the electron‐rich sulfur (S) atoms in BDTS‐2DPP as Lewis base can well passivate the under‐coordinated Pb in CH_3_NH_3_PbI_3_(Cl) without annealing. The PSCs with BDTS‐2DPP interlayer at the interface of perovskite/spiro‐OMeTAD show a higher PCE (18.2%) than the control devices without the BDTS‐2DPP interlayer (16.9%). After stored under ambient conditions (humidity of ≈40% and temperature of ≈25 °C) in the dark over 7 d, the PCEs of the unencapsulated PSCs without BDS‐2DPP decay dramatically to 30% of their initial values, while the devices with BDTS‐2DPP interlayer retain about 80% of their initial values, indicating that the BDTS‐2DPP interlayer enhances the device stability.

## 2 Results and Discussion

The energy levels of the components of the PSCs are shown in Figure 1b. The HOMO (−5.28 eV) and LUMO (−3.49 eV) levels of BDTS‐2DPP are calculated from cyclic voltammetry measurement. Obviously, the HOMO level of BDTS‐2DPP is higher than the valence band of CH_3_NH_3_PbI_3_(Cl), indicating a favorable hole transport from CH_3_NH_3_PbI_3_(Cl) to BDTS‐2DPP. Meanwhile, the LUMO level of BDTS‐2DPP is much higher than the conduction band of CH_3_NH_3_PbI_3_(Cl), which benefits the electron blocking. In addition, the hole mobility of BDTS‐2DPP is two orders of magnitude higher than that of spiro‐OMeTAD (≈10^−4^ cm^2^ V^−1^ s^−1^), which facilitates hole extraction. As a result, BDTS‐2DPP has the potential to work as an efficient hole extraction and transport layer in the PSCs.

**Figure 2:**
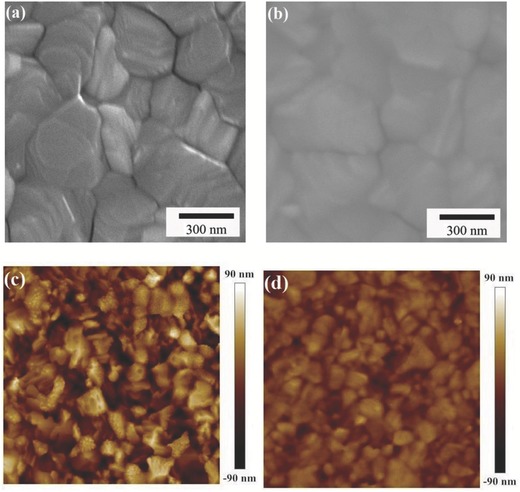
a) Top‐view SEM image of glass/TiO_2_/perovskite film, b) top‐view SEM image of glass/TiO_2_/perovskite/BDTS‐2DPP film, c) AFM height image of glass/TiO_2_/perovskite film, and d) AFM height image of glass/TiO_2_/perovskite/BDTS‐2DPP film. The scan sizes of all AFM height images are 5 µm × 5 µm.

The device structure and cross‐sectional scanning electron microscopy (SEM) image are shown in Figure 1c,d. The perovskite crystal structure was certified by X‐ray diffraction (XRD). As shown in Figure S1 (Supporting Information), the intense peak at 14.2° and 28.3° is assigned to the (110) and (220) plane of tetragonal phase perovskite, respectively, similar to the previous work.^13^ The cross‐sectional SEM image of the device confirms that the device configuration is a well‐defined layer‐by‐layer structure. The top‐view SEM images of the CH_3_NH_3_PbI_3_(Cl) film on TiO_2_ and the BDTS‐2DPP film on CH_3_NH_3_PbI_3_(Cl) are shown in **Figure** **2**a,b. The perovskite film is uniform but with relatively large roughness, and the surface appears smooth with complete coverage by the BDTS‐2DPP. The surface morphology can also be seen from the tapping mode atomic force microscopy (AFM) images (Figure 2c,d). The roughness is reduced from 28.4 to 13.5 nm after spin‐coating a thin layer of BDTS‐2DPP on perovskite.

**Figure 3:**
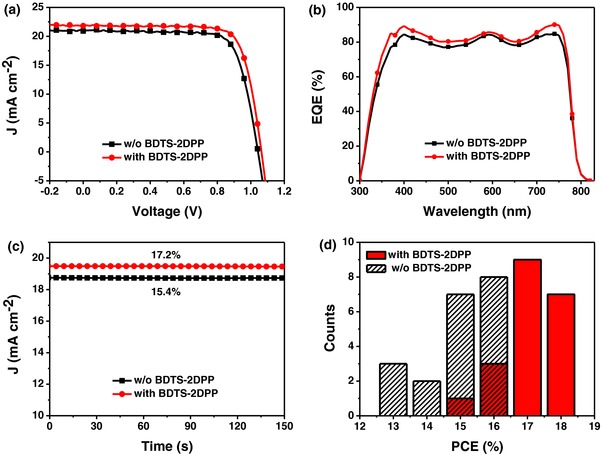
a) *J*–*V* curves and b) EQE spectra of PSCs without and with BDTS‐2DPP, c) steady‐state measurement of current and PCE of PSCs without and with BDTS‐2DPP, and d) histogram of efficiencies based on 20 devices.

To investigate the effect of BDTS‐2DPP on the performance of PSCs, HTLs of doped spiro‐OMeTAD without and with BDTS‐2DPP as the interlayer were used in a device with the structure of ITO/compact TiO_2_/CH_3_NH_3_PbI_3_(Cl)/HTL/Au. **Figure** **3**a shows the current density–voltage (*J*–*V*) curves of the optimized devices. The device with BDTS‐2DPP interlayer has a PCE of 18.2% with a high short circuit current density (*J*_SC_) of 22.0 mA cm^−2^, an open circuit voltage (*V*_OC_) of 1.06 V, and a FF of 77.9% (**Table** **1**), which is higher than that of the control devices without BDTS‐2DPP under the same conditions (16.9%). Figure 3b shows the external quantum efficiency (EQE) spectra of the devices. The perovskite solar cells using BDTS‐2DPP as the interlayer show stronger photocurrent response from 300 to 800 nm than the control devices without BDTS‐2DPP, resembling the trend in *J*_SC_. The integrated *J*_SC_ from the EQE spectra match the average *J*_SC_ well, while the average *J*_SC_ of PSCs with and without BDTS‐2DPP is 21.0 and 20.6 mA cm^−2^, respectively (Table 1). Figure 3c shows the steady‐state current output of the PSCs without and with BDTS‐2DPP interlayer under the maximum power point. The device with BDTS‐2DPP exhibits a stabilized efficiency of 17.2%, superior to that of the control device without BDTS‐2DPP (15.4%). Both of the stabilized efficiencies match well with those from *J*–*V* measurement. Moreover, the BDTS‐2DPP based PSCs show good reproducibility (Figure 3d). The *J*–*V* hysteresis behavior of PSCs is improved with the BDTS‐2DPP interlayer (Figure S2, Supporting Information).

**Table 1:** Performance parameters of the optimized PSCs at reverse voltage scan

Device	*J*_SC_ [mA cm^−2^]	Calculated *J*_SC_ [mA cm^−2^]	*V*_OC_ [V]	FF [%]	PCE [%]	
					Best	Average
w/o BDTS‐2DPP	21.3	20.7	1.04	75.9	16.9	16.0
With BDTS‐2DPP	22.0	21.5	1.06	77.9	18.2	17.1

**Figure 4:**
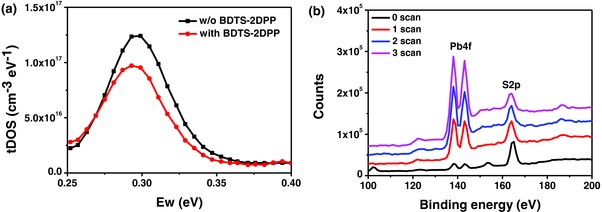
a) tDOS of the PSCs without and with BDTS‐2DPP and b) depth analysis XPS survey scans in different depth from the top surface of perovskite/BDTS‐2DPP film.

In order to investigate the interaction between BDTS‐2DPP interlayer and perovskite, we measured the trap density of the devices with and without BDTS‐2DPP interlayer to get the energetic profile of trap density of states (tDOS) (details in the Supporting Information).^14^ As shown in **Figure** **4**a, there is a relatively large density of defect states on the order of 1 × 10^17^ cm^−3^ eV^−1^ in the devices without BDTS‐2DPP interlayer. The tDOS of traps with an energy depth of 0.25–0.35 eV decreases by 20% after depositing BDTS‐2DPP layer on top of the perovskite film, indicating that the BDTS‐2DPP interlayer plays a role in defect passivation. From the dark *J*–*V* curves, the devices with BDTS‐2DPP show lower leakage current, indicating fewer defects than the control devices without BDTS‐2DPP (Figure S3, Supporting Information).

Meanwhile, we used depth analysis X‐ray photoelectron spectroscopy (XPS) to measure the ratio of atoms (S and Pb) and estimate the BDTS‐2DPP weight ratios penetrating into the perovskite layer (Figure 4b). Since only BDTS‐2DPP contains sulfur (S) and only perovskite contains lead (Pb), we attribute the S2p spectral line (≈164 eV) to BDTS‐2DPP and Pb4f spectral line (≈140 eV) to perovskite. One BDTS‐2DPP molecule contains ten S atoms, while one perovskite molecule contains one Pb atom. So that we can calculate the BDTS‐2DPP weight content from the Pb/S ratio (details in the Supporting Information). The weight ratio of BDTS‐2DPP at 0, 1, 2, and 3 scan is 93%, 64%, 50%, and 40%, respectively, indicating that BDTS‐2DPP can penetrate into perovskite layer to passivate defects (the scan number is proportional to the depth: “0 scan” means at the top surface of perovskite/BDTS‐2DPP film (depth = 0 nm), and the depth increases with the scan number increasing from 0 to 4).

**Figure 5:**
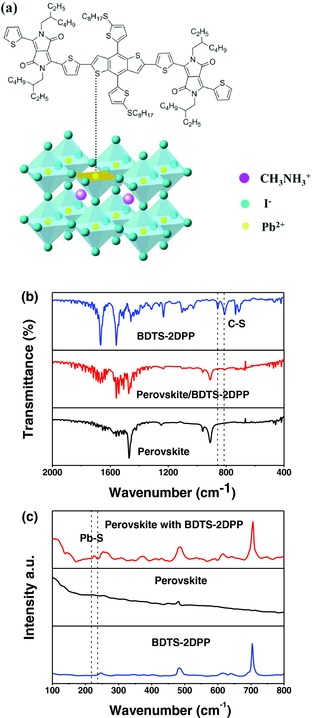
a) Schematic illustration of the potential surface defect sites of perovskite and the passivation effect of BDTS‐2DPP; b) FTIR spectra of the neat BDTS‐2DPP film, neat perovskite film, and perovskite/BDTS‐2DPP film; and c) Raman spectra of the neat BDTS‐2DPP film, neat perovskite film, and perovskite/BDTS‐2DPP film.

Fourier transform infrared (FTIR) spectroscopy is also employed to investigate the interaction between BDTS‐2DPP and perovskite (**Figure** **5**a). To distinguish the difference in the spectra between BDTS‐2DPP and perovskite/BDTS‐2DPP films, the spectra are normalized according to the peak of 1558 cm^−1^, which is assigned to the C=C stretching vibration and is thus insensitive to Pb ions. As shown in Figure 5b, for the neat BDTS‐2DPP film, the peaks at 856 and 809 cm^−1^ are assigned to the antisymmetric C—S stretching and symmetric C—S stretching modes, respectively.^15^ The FTIR spectrum of perovskite/BDTS‐2DPP film shows weaker ν(C—S) (856 and 809 cm^−1^), which confirms the presence of interaction between S and perovskite. Raman spectroscopy is further employed to analyze the interaction between BDTS‐2DPP and perovskite (Figure 5c). One new weak Raman band at 226 cm^−1^ is assigned to Pb—S stretching, which is a direct evidence of the formation of Lewis adduct between the under‐coordinated Pb in perovskite film and S in BDTS‐2DPP.^16^ It should be noted that the samples for FTIR and Raman spectra measurements were prepared at room temperature, indicating that the passivation of perovskite by BDTS‐2DPP is still efficient without thermal treatment.

**Figure 6:**
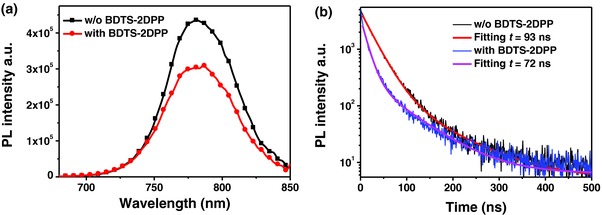
a) The steady‐state PL spectra and b) time‐resolved PL decay transient spectra of glass/perovskite/spiro‐OMeTAD and glass/perovskite/BDTS‐2DPP/spiro‐OMeTAD.

To further investigate the charge extraction, we measured the steady‐state photoluminescence (PL) and time‐resolved PL spectra of glass/perovskite/spiro‐OMeTAD and glass/perovskite/BDTS‐2DPP/spiro‐OMeTAD (**Figure** **6**a,b). From the steady‐state PL spectra, we observed a significant PL quenching of 30% when the perovskite/spiro‐OMeTAD interface is modified with the BDTS‐2DPP layer, indicating efficient charge extraction. The time‐resolved PL decay was measured with the peak emission at 780 nm as shown in Figure 6b. Fitting the data with two‐component exponential decay (here, the longer lifetime was used for comparison) yields the lifetime of carriers. The PL decay of glass/perovskite/spiro‐OMeTAD exhibits a PL lifetime of 93 ns, while the PL lifetime is shortened to 72 ns with the BDTS‐2DPP interlayer, indicating better charge extraction ability of BDTS‐2DPP/spiro‐OMeTAD compared with neat spiro‐OMeTAD.

**Figure 7:**
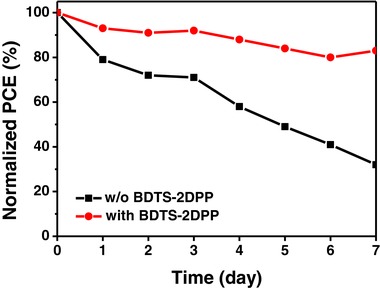
Stability tests of the PSCs under ambient conditions.

The stability tests of PSCs with and without BDTS‐2DPP were performed under ambient conditions (humidity of ≈40% and temperature of ≈25 °C) with ten full devices without encapsulation, and the average data are shown in **Figure** **7**. The unencapsulated solar cells were stored under ambient conditions in the dark. The PCEs of the PSCs without BDTS‐2DPP decay dramatically to 30% of their initial values over 7 d, while the devices with BDTS‐2DPP interlayer retain about 80% of their initial PCEs during the same test period. The reason for the obvious difference in the device stability can be attributed to the hydrophobicity of BDTS‐2DPP. The water contact angles of dopant‐free BDTS‐2DPP and doped spiro‐OMeTAD are 90.8° and 74.2°, respectively (Figure S4, Supporting Information). Compared with the single doped spiro‐OMeTAD film, film with BDTS‐2DPP interlayer efficiently prevents water from penetrating into the perovskite layer, resulting in much slower degradation of the perovskite material.

## 3 Conclusion

We demonstrate a facile and efficient interfacial engineering strategy using a p‐type organic semiconductor BDTS‐2DPP at the perovskite/HTL interface in PSCs for the first time. Steady‐state and time‐resolved PL spectra reveal that the BDTS‐2DPP interlayer is beneficial to efficient hole extraction from perovskite film and transfer to HTL due to high hole mobility and an appropriate energy level alignment to CH_3_NH_3_PbI_3_(Cl). Moreover, the electron‐rich S atoms in BDTS‐2DPP can act as Lewis base to passivate the trap states of the perovskite layer through the formation of Lewis adducts of Pb and S, which is confirmed by FTIR, Raman spectra, and tDOS measurements. The superhydrophobic BDTS‐2DPP layer behaves as an encapsulation layer to protect the perovskite film against the moisture. The PSCs with BDTS‐2DPP interlayer exhibit a significantly enhanced efficiency and better stability than the control devices without BDTS‐2DPP, due to the better hole extraction, defect passivation, and protection of the perovskite material from moisture. These results point a promising direction toward further development of high‐efficiency and high‐stability PSCs.

## 4 Experimental Section

*Materials*: The molecule BDTS‐2DPP was synthesized according to the published procedure.[[qv: 12a]] Unless stated otherwise, all reagents were obtained commercially and were used without further purification.

*Device Fabrication*: PSCs were fabricated with a structure of ITO/compact TiO_2_/CH_3_NH_3_PbI_3_(Cl)/HTL/Au. The indium tin oxide (ITO) glass was precleaned in an ultrasonic bath of detergent water, ultrapure water, acetone, and isopropanol. A 40 nm thick layer of a TiO_2_ nanoparticle in ethanol (5.3 mg mL^−1^) with the appropriate amount of titanium diisopropoxide bis(acetylacetonate) (TiAcAc) was spin‐coated onto an ITO glass substrate and annealed at 150 °C for 30 min in air. Next, the substrates were transferred into a nitrogen‐filled glove box. A solution of PbI_2_ (dissolved in DMF, 450 mg mL^−1^) was then spin‐coated on ITO/TiO_2_ substrate at 3000 rpm for 30 s and annealed at 90 °C for 10 min. The mixture of CH_3_NH_3_I (50 mg mL^−1^) and CH_3_NH_3_Cl (5 mg mL^−1^) dissolved in isopropanol was spin‐coated onto the dried PbI_2_ layer at 3000 rpm for 30 s. Then, the obtained films were annealed at 150 °C for 15 min in air. The dopant‐free BDTS‐2DPP interlayer was spin‐coated onto the perovskite layer at 3000 rpm for 30 s with a concentration of 10 mg mL^−1^ in chlorobenzene without any annealing treatment. The doped spiro‐OMeTAD was spin‐coated at 3000 rpm for 30 s at a concentration of 80 mg mL^−1^ with the addition of 35 µL Li‐TFSI/acetonitrile (260 mg mL^−1^) and 28 µL 4‐*tert*‐butylpyridine. Finally, an 80 nm gold layer was deposited on the top of HTL through shadow masks via thermal evaporation under high vacuum (≈2 × 10^−6^ Torr).

*Measurements*: The current density−voltage (*J−V*) characteristics of photovoltaic devices were obtained using a Keithley 2400 source‐measure unit under simulated sunlight from an Oriel 300 solar simulator, and the light intensity was calibrated using a KG‐5 Si diode. The effective area of each cell was 10.2 mm^2^ defined by masks for all the photovoltaic devices discussed in this work. The EQE spectrum was measured using a Solar Cell Spectral Response Measurement System QE‐R3011 (Enlitech Co., Ltd). The light intensity at each wavelength was calibrated using a standard single crystal Si photovoltaic cell. The morphology was measured using a scanning electron microscope (Hitachi S4800). AFM images were obtained by a Multimode 8 scanning probe microscopy (Bruker). The XRD spectra were obtained from a D/MAX 2400 diffractometer with Cu Kα radiation (Rigaku). FTIR spectra were obtained by a TENSOR27 (Bruker), and Raman spectra were obtained by a LabRAM HR Evolution spectrometer (Horiba). TAS was performed using an IM6ex electrochemical workstation (Zahner), in which the scanning frequency was set between 0.1 and 10^6^ Hz, and the amplitude of the sine perturbation bias was set to 10 mV. XPS was performed on the Thermo Scientific ESCALab 250Xi using 200 W monochromated Al Kα radiation. The 500 µm X‐ray spot was used for XPS analysis. The base pressure in the analysis chamber was about 3 × 10^−10^ mbar. Typically, the hydrocarbon C1s line at 284.8 eV from adventitious carbon was used as an energy reference. Steady‐state PL and time‐resolved transient‐state PL was measured by FLS980 (Edinburgh Instruments Ltd.) with an excitation at 470 nm. Static contact angles were measured on a Dataphysics OCA20 contact‐angle system at ambient temperature (the test liquid is water). All the measurements of the solar cells were performed under ambient atmosphere at room temperature without encapsulation.

## Supporting information

Supplementary
