# Supplementary material for: High‐Mobility p‐Type Organic Semiconducting Interlayer Enhancing Efficiency and Stability of Perovskite Solar Cells
Source: Adv Sci (Weinh). 2017 Apr 21;4(9):1700025. doi: 10.1002/advs.201700025 (PMC5604372; doi:10.1002/advs.201700025)
Supplement: Supplementary file 1 — Supplementary [file ADVS-4-na-s001.pdf]

Copyright WILEY-VCH Verlag GmbH & Co. KGaA, 69469 Weinheim, Germany, 2017.

ADVANCED SCIENCE Open Access

Supporting Information

for *Adv. Sci.,* DOI: 10.1002/advs.201700025

High-Mobility p-Type Organic Semiconducting Interlayer Enhancing Efficiency and Stability of Perovskite Solar Cells

Mingyu Zhang, Jiayu Wang, Liang Li, Guanhaojie Zheng, Kuan Liu, Meng Qin, Huanping Zhou, and Xiaowei Zhan**

Supporting Information

High-Mobility p-Type Organic Semiconducting Interlayer Enhancing Efficiency and Stability of Perovskite Solar Cells

Mingyu Zhang, Jiayu Wang, Liang Li, Guanhaojie Zheng, Kuan Liu, Meng Qin, Huanping Zhou,* Xiaowei Zhan*

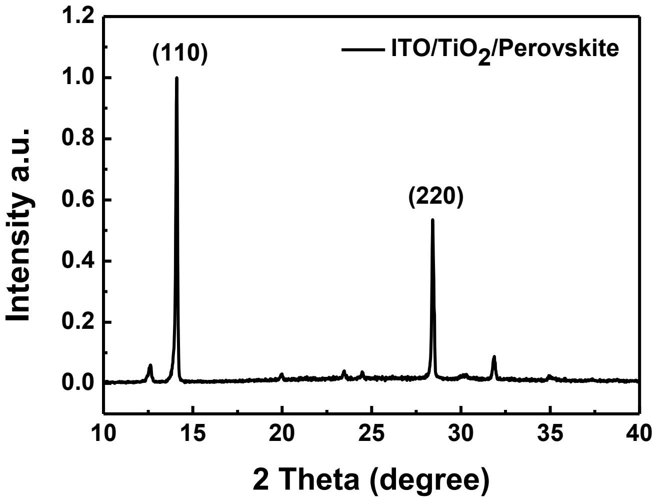

Figure S1. XRD pattern of perovskite film on ITO/TiO_2 substrate

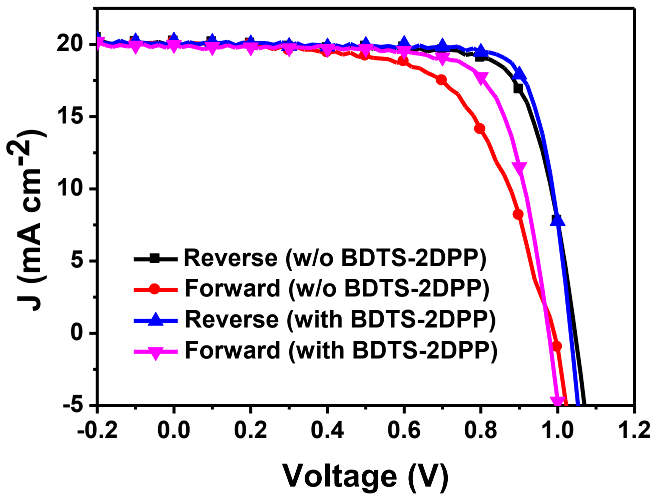

Figure S2. *J-V* hysteresis curves of PSCs without and with BDTS-2DPP.

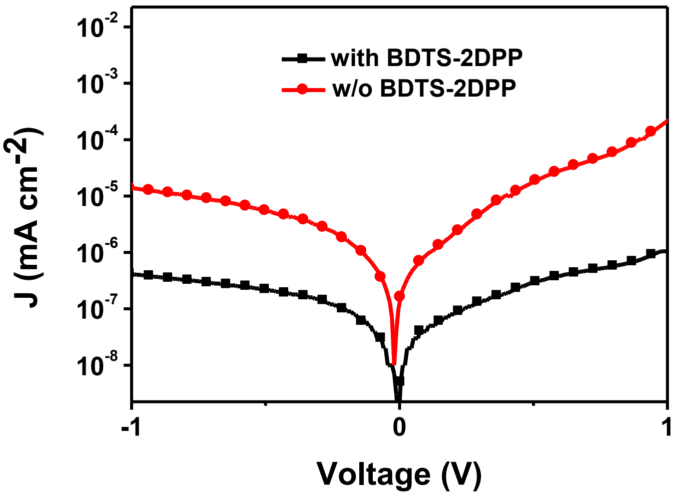

Figure S3. Dark *J-V* curves of the PSCs without and with BDTS-2DPP.

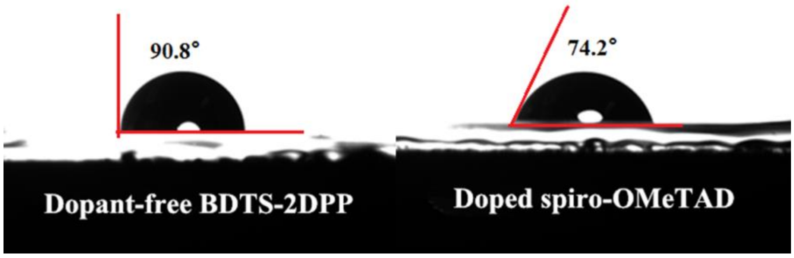

Figure S4. Water contact angles of different HTL films on glass substrate

Details for the tDOS

The thermal admittance spectroscopy (TAS) method was applied to analyze the reduction of trap states in perovskite films by the passivation of the BDTS-2DPP film. The energetic profile of trap density of states (tDOS) can be derived from the angular frequency dependent capacitance using the equation:

$$N_{\mathrm{T}}(E_{\omega}) = -\frac{V_{\mathrm{bi}}}{qW}\frac{dC}{d\omega}\frac{\omega}{k_{\mathrm{B}}T}$$

Where C is the capacitance, ω is the angular frequency, q is the elementary charge, k_{B} is the Boltzmann's constant and T is the temperature. V_{bi} and W are the built-in potential and depletion width, respectively, which were extracted from the Mott–Schottky analysis. The applied angular frequency ω defines an energetic demarcation:

$$E_{\omega} = k_{\mathrm{B}}T\ln(\frac{\omega_0}{\omega})$$

Where ω_0 is the attempt-to-escape frequency. The trap states below the energy demarcation can capture or emit charges with the given ω and contribute to the capacitance.

Calculation of the surface composition

BDTS-2DPP weight content

$$= \frac{\mathrm{BDTS-2DPP\ weight}}{\mathrm{BDTS-2DPP\ weight+perovskite\ weight}}$$

$$= \frac{\mathrm{n(BDTS-2DPP)M(BDTS-2DPP)}}{\mathrm{n(BDTS-2DPP)M(BDTS-2DPP)+n(perovskite)M(perovskite)}}$$

$$= \frac{1}{1+\frac{\mathrm{n(perovskite)M(perovskite)}}{\mathrm{n(BDTS-2DPP)M(BDTS-2DPP)}}}$$

$$\frac{\mathrm{Pb}}{\mathrm{S}} = \frac{\mathrm{n(perovskite)}}{\mathrm{10n(BDTS-2DPP)}}$$

$$\frac{\mathrm{n(perovskite)}}{\mathrm{n(BDTS-2DPP)}} = \frac{\mathrm{10Pb}}{\mathrm{S}}$$

BDTS-2DPP weight content = $\frac{1}{1+\frac{10\text{Pb}}{\text{S}}*\frac{\text{M(perovskite)}}{\text{M(BDTS}-\text{2DPP)}}} = \frac{1}{1+\frac{10\text{Pb}}{\text{S}}*0.37} = \frac{1}{1+\frac{3.7\text{Pb}}{\text{S}}}$

n(BDTS-2DPP) is the mole number of BDTS-2DPP;

n(perovskite) is the mole number of perovskite;

M(BDTS-2DPP) is the molecular weight of BDTS-2DPP (1686.6);

M(perovskite) is the molecular weight of perovskite (619.9);

$\frac{\text{Pb}}{\text{S}}$ is the atom ratio of Pb/S.
